# A Novel WRKY Transcription Factor HmoWRKY40 Associated with Betalain Biosynthesis in Pitaya (*Hylocereus monacanthus*) through Regulating *HmoCYP76AD1*

**DOI:** 10.3390/ijms22042171

**Published:** 2021-02-22

**Authors:** Lulu Zhang, Canbin Chen, Fangfang Xie, Qingzhu Hua, Zhike Zhang, Rong Zhang, Jianye Chen, Jietang Zhao, Guibing Hu, Yonghua Qin

**Affiliations:** State Key Laboratory for Conservation and Utilization of Subtropical Agrobioresources/Guangdong Provincial Key Laboratory of Postharvest Science of Fruits and Vegetables/Key Laboratory of Biology and Genetic Improvement of Horticultural Crops (South China), Ministry of Agriculture and Rural Affairs, College of Horticulture, South China Agricultural University, Guangzhou 510642, Guangdong, China; 18848966689@163.com (L.Z.); nnchencanbin@163.com (C.C.); Xiefangfang202012@163.com (F.X.); huaqingzhu@stu.scau.edu.cn (Q.H.); poloky2@163.com (Z.Z.); r-zhang@scau.edu.cn (R.Z.); chenjianye@scau.edu.cn (J.C.); jtzhao@scau.edu.cn (J.Z.); guibing@scau.edu.cn (G.H.)

**Keywords:** pitaya, betalain biosynthesis, transcription factor, HmoWRKY40, VIGS

## Abstract

Betalains are water-soluble nitrogen-containing pigments with multiple bioactivities. Pitaya is the only large-scale commercially grown fruit containing abundant betalains for consumers. However, the upstream regulators in betalain biosynthesis are still not clear. In this study, HmoWRKY40, a novel WRKY transcription factor, was obtained from the transcriptome data of pitaya (*Hylocereus monacanthus*). HmoWRKY40 is a member of the Group IIa WRKY family, containing a conserved WRKY motif, and it is located in the nucleus. The betalain contents and expression levels of HmoWRKY40 increased rapidly during the coloration of pitaya and reached their maximums on the 23rd day after artificial pollination (DAAP). Yeast one-hybrid and transient expression assays showed that HmoWRKY40 could bind and activate the promoter of *HmoCYP76AD1*. Silencing the *HmoWRKY40* gene resulted in a significant reduction of betacyanin contents. These results indicate that HmoWRKY40 transcriptionally activates *HmoCYP76AD,* which is involved in the regulation of pitaya betalain biosynthesis. The results of the present study provide new regulatory networks related to betalain biosynthesis in pitaya.

## 1. Introduction

Betalains are water-soluble and nitrogen-containing pigments with antioxidant properties, which were first extracted from the common beet (*Beta vulgaris*). Betalains can be divided into betacyanins and betaxanthins according to their chemical structures [1]. Betalains are restricted to 13 families of the order Caryophyllales, except thatMolluginaceae and Caryophyllaceae produce anthocyanins instead of betalain pigments. In nature, betalains and anthocyanins are mutually exclusive and cannot co-exist in one plant at the same time. Therefore, betalains can be used as additional help for classification in the plant kingdom [2].

Betalains not only can present different colors but also play fundamental roles in plant responses and adaptation to biotic and abiotic stresses, such as disease, drought, high salinity and low- and high-temperature stresses [3,4,5]. Betalains have a high nutritional value and are widely used as natural colorants of foods, drugs and cosmetics due to their high antioxidative and free radical scavenging activities [6,7,8]. Therefore, understanding the biosynthetic and regulatory pathways for betalain biosynthesis is essential to the improvement of betalain production in plants through biotechnological approaches. Betalains are secondary metabolites derived from the amino acid L-tyrosine. Cytochrome P450-76AD1 (CYP76AD1) or tyrosinase (TYR) catalyzes the conversion of tyrosine to L-3,4-dihydroxyphenylalanine (L-DOPA). DOPA 4,5-dioxygenase (DOD) converts L-DOPA to betalamic acid. Betaxanthins and betacyanins are produced through the spontaneous conjugation of betalamic acid with amines or with L-DOPA derivatives, respectively. Glucosyltransferases (GTs) catalyze 5-O glucosylation of cyclo-DOPA or, alternatively, 5-O or 6-O glucosylation of betanidin [9,10,11,12,13]. In addition to the key genes, the MYB and WRKY transcription factors (TFs) are also reported to be involved in the regulation of betalain biosynthesis [14,15,16].

A MYB-family TF (BvMYB1) involved in betalain biosynthesis was obtained from *B. vulgaris*. Overexpression of 35S::BvMYB1 in white beet resulted in the production of a red transgenic fiber ‘hairy’ root, indicating that *BvMYB1* can regulate the betalain biosynthesis in *B. vulgaris* [15]. This is the first MYB TF reported to be involved in the regulation of betalain biosynthesis. WRKYs are reported to regulate plant metabolite biosynthesis, such as phenylpropanoids, alkaloids and terpenes, by regulating metabolite biosynthetic genes. HmoWRKY44, a WRKY TF, could regulate betalain biosynthesis in pitaya through transcriptionally activating the promoter of *HmoCYP76AD1* [16]. This is the first report of WRKY TFs being involved in the betalain biosynthesis of pitaya. These results suggest that MYB and WRKY TFs play significant roles in betalain biosynthesis by regulating the betalain biosynthetic genes. However, the regulatory pathway for betalain biosynthesis remains to be fully clarified.

Pitaya, also known as dragon fruit, is one of the important tropical fruits in the *Hylocereus* of the Cactaceae family [17]. Pitayas contain a large amount of betalains. Recently, we isolated several key structural genes, such as *HmoCYP76AD1*, *HmB5GT1* and *HmHCGT2,* involved in the betalain biosynthetic pathways from transcriptomic data [10,13]. In this study, we investigated the possible association of HmoWRKY40, a Group IIa WRKY TF, with the direct activation of *HmoCYP76AD1,* which is responsible for betalain biosynthesis in pitayas. The present results provide new information on the transcriptional regulation of betalain production in pitaya.

## 2. Results

### 2.1. Changes in Betalain Contents at All Coloration Stages of Pitaya Pulp

**Figure 1:**
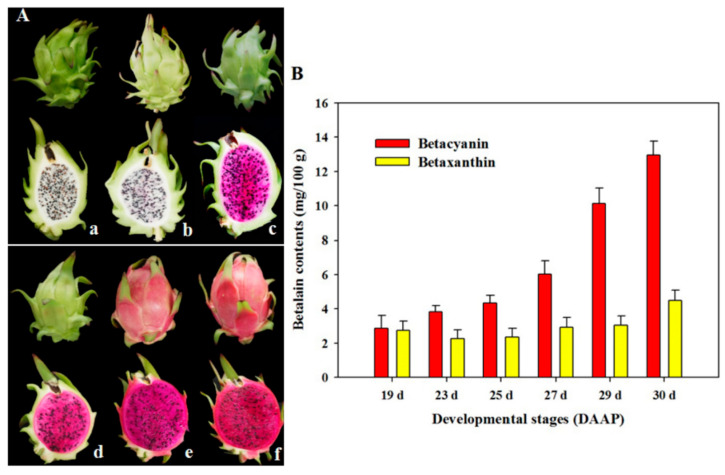
Fruits and betalain contents at the different developmental stages of the Guanhuahong pitaya: (**A**) Fruits at the different developmental stages of the Guanhuahong pitaya. a–f, fruits 19, 23, 25, 27, 29 and 30 days after artificial pollination (DAAP) and (**B**) Betalain contents in the pulps of the different developmental stages of the Guanhuahong pitaya. Three independent experiments were conducted (n = 3). The error bars indicate one standard error.

The contents of betacyanins and betaxanthins were detected in the pulps of the Guanhuahong pitaya during fruit maturation. As shown in Figure 1, the contents of betacyanins gradually increased at all stages of pulp coloration in the Guanhuahong pitaya. Higher contents of betacyanins were detected compared with betaxanthins. Betacyanins increased during fruit maturation and the highest content of betacyanins was detected at the fully mature stage, while betaxanthins kept lower contents at all stages in the Guanhuahong pitaya (Figure 1B).

### 2.2. Promoter Analyses of HmoCYP76AD1

*CYP76AD1*, encoding a novel cytochrome P450, is a key gene for the conversion of L-DOPA to cyclo-DOPA, which produces the red betacyanin pigment in beets [9]. Our previous studies showed that *HmoCYP76AD1* is likely involved in betalain biosynthesis [10]. Putative cis-acting elements in the promoter regions of *HmoCYP76AD1* (the sequence is listed in the Supplementary Text S1) were analyzed using the Plant-CARE database (Table 1). The locations of motifs are shown based on “+1” being the transcription start site. The core promoter element of “TATA-box” motifs is around −30 of the transcription start, which was proven for most of the promoters (plants and other eukaryotic nuclear genes). “TATA-box” motifs in the promoter of the *HmoCYP76AD1* ranged from 130 bp downstream (+) to 730 bp upstream (−). These results indicate that the promoter of *HmoCYP76AD1* is a variant promoter. In addition to the core cis-acting elements, such as a TATA-box and a CAAT-box, a typical W-box motif with a core sequence of (C/T)TGAC(C/T) was identified in the *HmoCYP76AD1* promoter (Table 1). The W-box motif of *HmoCYP76AD1* was predicted to be at 120 bp (−) upstream. The W-box is a cognate binding site for WRKY TFs, suggesting the possible involvement of WRKY TFs in the regulation of *HmoCYP76AD1*.

### 2.3. Cloning and Sequence Analyses of HmoWRKY40

**Figure 2:**
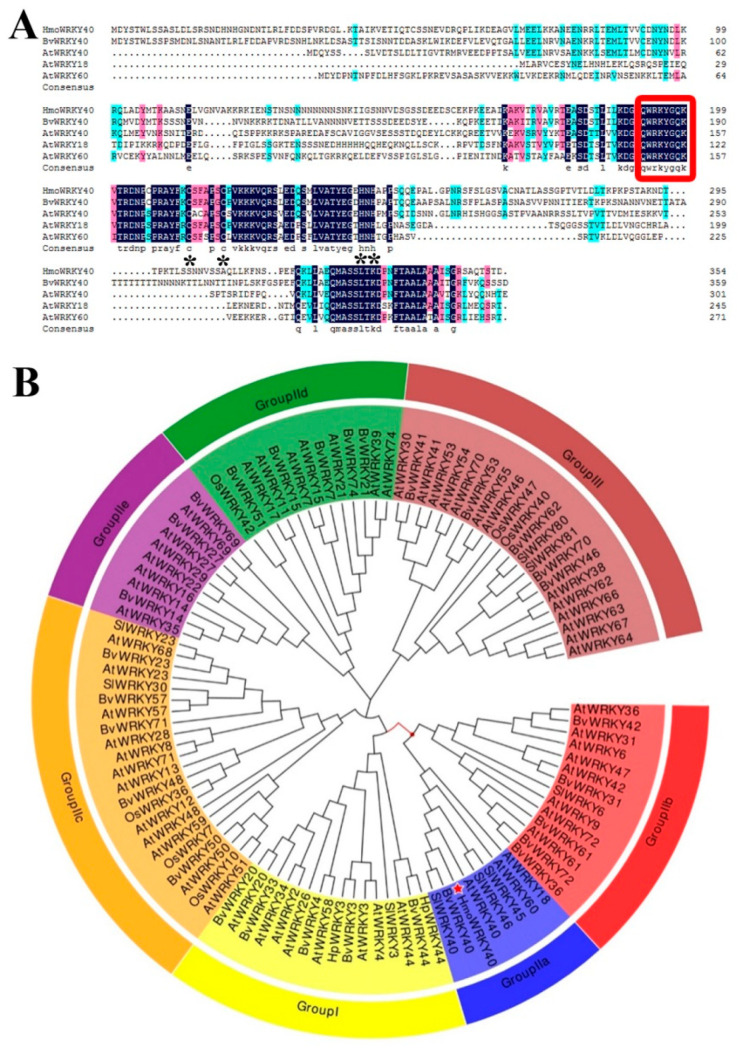
Bioinformatic analyses of HmoWRKY40: (**A**) Multiple alignment of HmoWRKY40 with the other plant WRKYs. The same and similar amino acids are indicated by blue and red shades, respectively. The WRKY motif and the zinc-finger structure are indicated by the red box and the black asterisks, respectively, and (**B**) The phylogenetic tree of *HmoWRKY40* and *WRKYs* from *Arabidopsis thaliana*, *Oryza sativa*, *Beta vulgaris* and *Solanum lycopersicum*. HmoWRKY40 protein is indicated by a red asterisk. The phylogenetic tree was constructed using the maximum likelihood method by MEGA7.0.

A full-length *WRKY* gene that was up-regulated during the color development of the pitaya fruit pulp was obtained from the RNA-Seq database of the Guanhuahong pitaya. The *WRKY* gene shows high similarity (50%) to *BvWRKY40* from *B. vulgaris* and therefore was named *HmoWRKY40* (the sequence is listed in Supplementary Text S2). The open reading frame (ORF) of the *HmoWRKY40* was 1065 bp in length and encoded a polypeptide of 354 amino acids, with a calculated molecular weight of 38.78 kDa and a theoretical isoelectric point (pI) of 6.91. *HmoWRKY40* had one highly conserved amino acid sequence of WRKYGQK at the N-terminal end, which is a WRKY domain and a defining characteristic of WRKY TFs. The HmoWRKY40 also contained a putative zinc-finger motif (C–X_4–5_–C–X_22–23_–H–X_1_–H) (Figure 2A). A phylogenetic tree was constructed using the amino acid sequence of *HmoWRKY40* and *WRKYs* from *Arabidopsis thaliana*, *Oryza sativa*, *B. vulgaris* and *Solanum lycopersicum*. *HmoWRKY40* was clustered with Group IIa WRKYs, along with the *BvWRKY40* from *B. vulgaris* and the *AtWRKY40*, *AtWRKY18* and *AtWRKY60* from *A. thaliana* (Figure 2B).

### 2.4. Expression Analyses of HmoWRKY40 and HmoCYP76AD1

**Figure 3:**
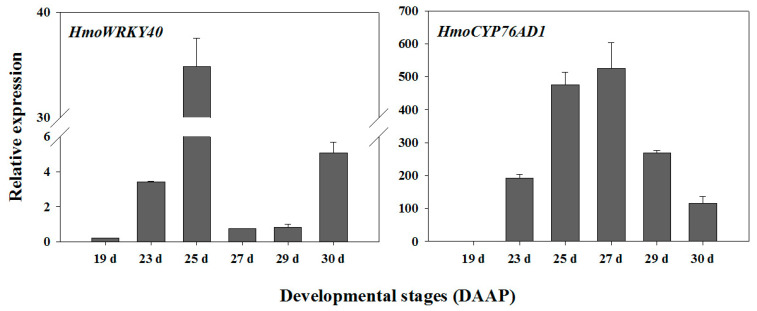
Expression analyses of *HmoWRKY40* and *HmoCYP76AD1* at all pulp coloration stages of the Guanhuahong pitayas. The expression level of the 19th DAAP was used as the calibrator (set as 1). The data represent the mean values from three biological replicates (±S.D.).

Expression levels of *HmoWRKY40* and *HmoCYP76AD1* were analyzed by RT-qPCR to explore their relationship to betalain biosynthesis during the different developmental stages of the Guanhuahong pitaya pulps (sequences are listed in Supplementary Text S2). Expression levels of *HmoWRKY40* and *HmoCYP76AD1* increased significantly during the coloration period (23rd to 25th DAAP) of the Guanhuahong pitaya pulps (Figure 3), which is consistent with the betalain accumulation and the color development of the pulps (Figure 1).

### 2.5. Nuclear Localization of HmoWRKY40

**Figure 4:**
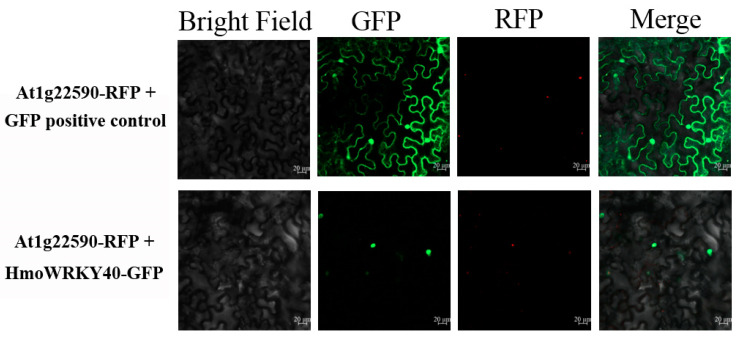
The subcellular location of *HmoWRKY40* in the leaves of *Nicotiana benthamiana*. *Agrobacterium tumefaciens* harboring HmoWRKY40-GFP and a GFP positive control were separately co-expressed with At1g22590-RFP (for nuclear positioning) into *N. benthamiana* leaves. The transient expression assays were repeated at least three times. Transient expressions of GFP and RFP signals were observed with a laser confocal microscope after 2 d of infiltration. Bars = 20 μm.

The full-length coding sequence of *HmoWRKY40* was fused with the *GFP* gene to analyze its subcellular localization. Transient expression of constructs in epidermal cells of *Nicotiana benthamiana* showed that the fluorescence of *HmoWRKY40* was detected exclusively in the nucleus, whilst for the positive control, the *GFP* signal was observed around the cytoplasm and the nucleus (Figure 4).

### 2.6. Analyses of Transcriptional Activities of HmoWRKY40

**Figure 5:**
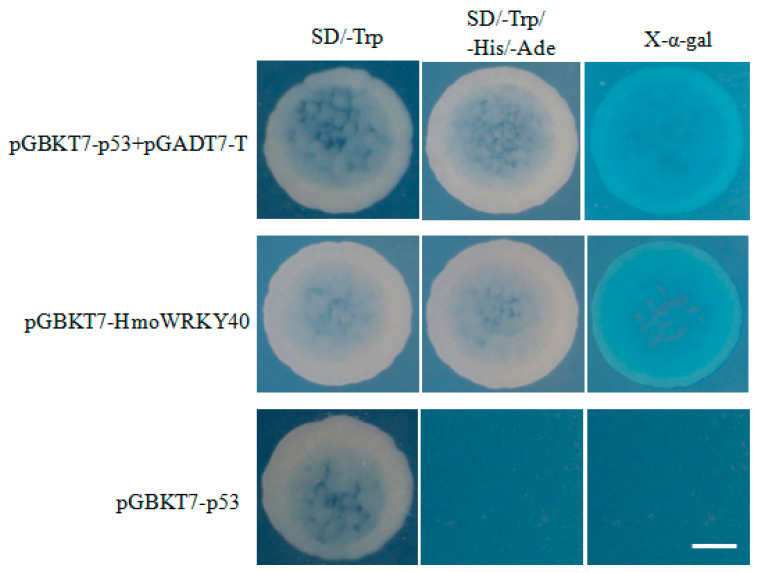
Transcriptional activation analyses of HmoWRKY40 in yeast cells. The coding region of HmoWRKY40 was inserted into the pGBKT7. The yeast cells of strain Y2HGold, harboring the pGBKT7-HmoWRKY40 plasmids, were cultivated on the SD/-Trp plate or the SD/-Trp-His-Ade plate for 3–5 d at 30 °C, followed by the α-galactosidase assay. The pGBKT7 and pGBKT7-53+pGADT7-T were used as the negative and positive control, respectively. Bars = 2 mm.

The transcriptional activities of HmoWRKY40 were determined according to the changes in the yeast color after staining. As shown in Figure 5, Y2H Gold yeast strains containing pGBKT7-HmoWRKY40, a positive control (pGBKT7-p53+pGADT7-T) and a negative control (pGBKT7) could all grow normally on the SD/-Trp medium. On the auxotrophic selection medium SD/-Trp-Ade-His, the yeast strains harboring both pGBKT7-HmoWRKY40 and the positive control all grew normally at 30 °C after 3 days of culturing, and the colonies turned blue with the addition of X-α-gal. However, the yeast strains harboring the empty pGBKT7 vector (the negative control) could not grow on the deficient medium SD/-Trp-Ade-His. These results suggest that HmoWRKY40 has a transcriptional activation function in yeast.

### 2.7. Virus-Induced Gene Silencing (VIGS) Analyses of HmoWRKY40

**Figure 6:**
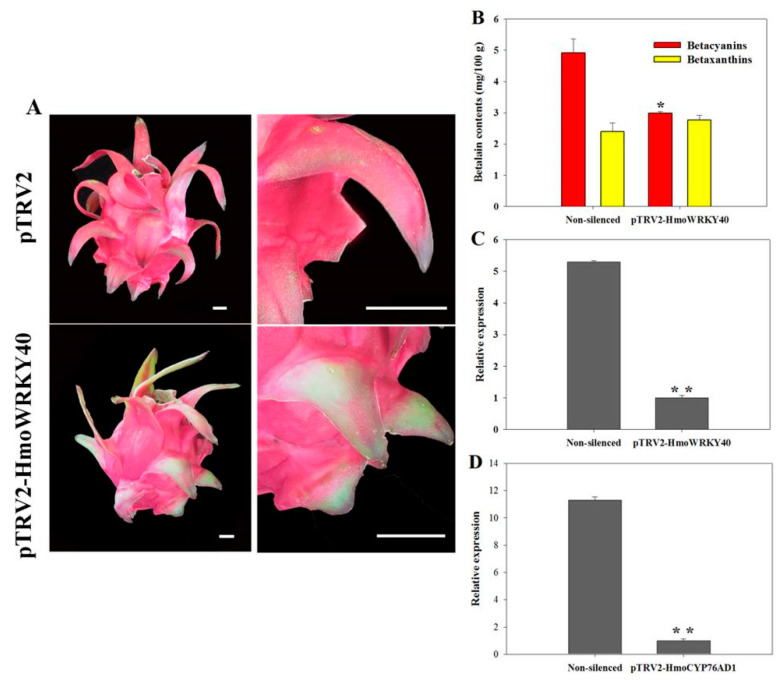
Silencing of *HmoWRKY40* inhibits betalain production: (**A**) Virus-induced gene silencing of *HmoWRKY40* in red scales. Bars = 2 cm, (**B**) Betalain contents in pitaya scales after virus-induced silencing of HmoWRKY40 (* indicates *p* < 0.05). Three independent experiments were conducted (n = 3). The error bars indicate one standard error, (**C**) RT-qPCR analyses of *HmoWRKY40* in virus-induced gene silencing (VIGS) treatment scales. The expression level of pTRV2-HmoWRKY40 was used as the calibrator (set as 1). The data represent mean values from three biological replicates (±S.D.). ** indicates significant differences at *p* value < 0.01 using a two-tailed *t*-test and (**D**) RT-qPCR analyses of *HmoCYP76AD1* in VIGS treatment scales. The expression level of pTRV2-HmoCYP76AD1 was used as the calibrator (set as 1).The data represent mean values from three biological replicates (±S.D.). ** indicates significant differences at *p* value < 0.01 using a two-tailed *t*-test.

A gene silencing assay was performed to further explore the function of *HmoWRKY40*. As shown in Figure 6A, when compared to the control, the silencing of *HmoWRKY40* exhibited green and yellow patches in the scales of the Hongguan No. 1 pitaya (*H. monacanthus*) and resulted in the reduction of betacyanins and betaxanthins (Figure 6B). Results from the RT-qPCR analyses showed that the *HmoWRKY40* and *HmoCYP76AD1* were silenced (Figure 6C,D). These results indicate that *HmoWRKY40* plays an important role in the betalain biosynthetic pathway of pitayas.

### 2.8. Interaction of HmoWRKY40 with the W-Box in the Promoter of HmoCYP76AD1

**Figure 7:**
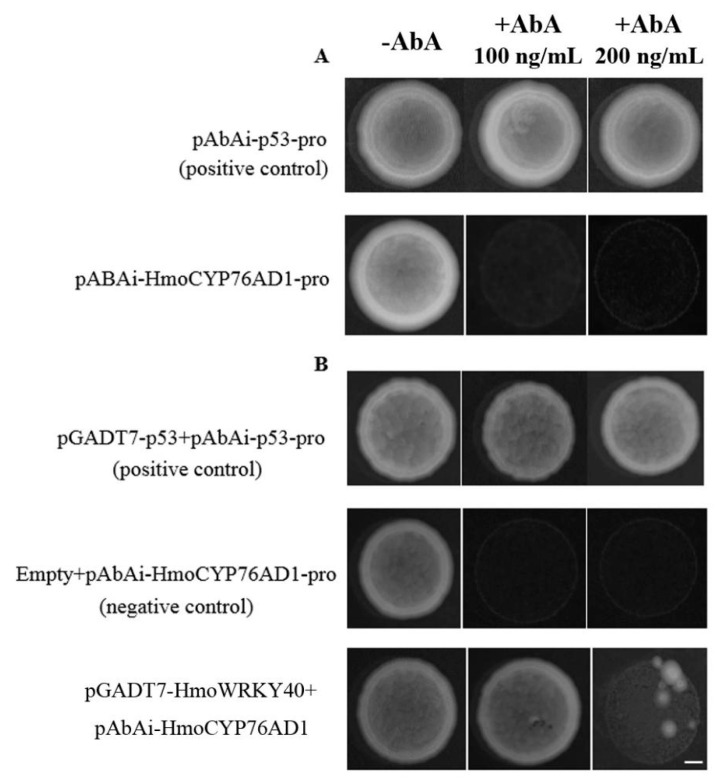
The relationship between HmoWRKY40 and the promoter region of *HmoCYP76AD1* by a yeast one-hybrid assay. Bars = 1 mm. (**A**) Self-activation assay of the *HmoCYP76AD1A* promoters. Yeast cells containing pAbAi-HmoCYP76AD1A-pro transformants were cultured on the SD/-Ura medium supplemented with different concentrations of AbA and (**B**) HmoWRKY40 interacted with HmoCYP76AD1A. Yeast cell transformants of HmoWRKY40 with pAbAi-HmoCYP76AD1A-pro were tested on SD/Leu of AbA at a concentration of 100 and 200 ng/mL. Yeast cells transformed with pGADT7-p53+pAbAi-p53 and pAbAi-HmoCYP76AD1A+pGADT7 were used as the positive and negative control, respectively.

The WRKY TF can recognize and bind to the W-box element in the promoter region of target genes. The promoter region of *HmoCYP76AD1* had the W-box element (Table 1). Therefore, the interaction between *HmoWRKY40* and the W-box in the promoter region of *HmoCYP76AD1* was analyzed using a yeast one-hybrid assay. Yeast cells harboring pABAi-HmoCYP76AD1-pro could not grow on the SD/-Ura medium supplemented with 100 or 200 ng/mL AbA (Figure 7A). Yeast cells containing pABAi-HmoCYP76AD1-pro+pGADT7-HmoWRKY40 grew on the SD/-Leu medium with the addition of 100 or 200 ng/mL AbA (Figure 7B). These results indicate that the HmoWRKY40 protein has a DNA binding activity and could directly bind to the promoter of *HmoCYP76AD1,* which is possibly involved in the betalain biosynthesis of pitaya.

### 2.9. HmoWRKY40 Activates the Transcription of Promoter of HmoCYP76AD1

**Figure 8:**
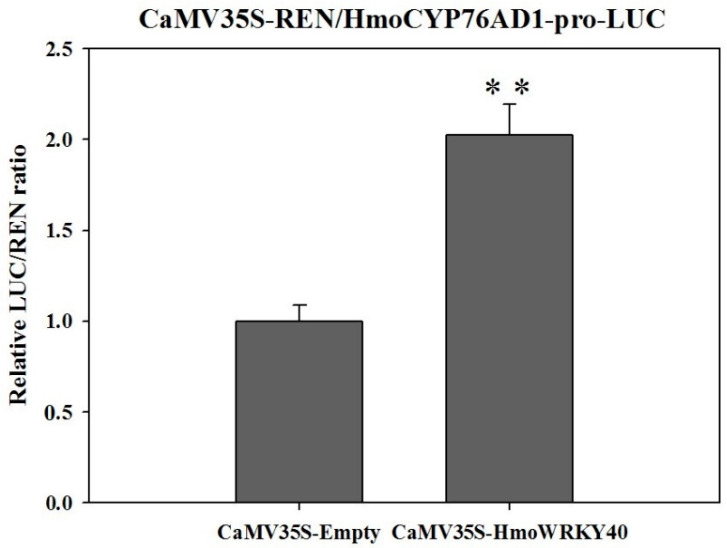
HmoWRKY40 activated the transcriptions of HmoCYP76AD1. The activation was measured as a ratio of LUC to REN. The ratio of LUC/REN of the empty vector plus promoter was used as the calibrator (set as 1). Three independent experiments were conducted (n  =  3). The error bars indicate one standard error. Significant differences from CaMV35S-Empty were determined according to Student’s *t*-test (** indicates *p* < 0.01).

The ability of HmoWRKY40 to activate the transcription of *HmoCYP76AD1* was performed using the transient dual-luciferase assays in tobacco leaves. As shown in Figure 8, the overexpression of HmoWRKY40 significantly increased the LUC/REN ratio. The LUC/REN ratio of CaMV35S-HmoWRKY40 co-transduced with CaMV35S-REN/HmoCYP76AD1-pro and was 2-fold higher than that of the control (Figure 8). These results demonstrate that HmoWRKY40 activates the transcription of the *HmoCYP76AD1* promoter, suggesting that HmoWRKY40 has a functional role in the regulation of *HmoCYP76AD1*.

## 3. Discussion and Conclusions

Betalains are one of the major plant pigments found only in some plants in the order of Caryophyllales, with the exception of the Caryophyllaceae and Molluginaceae families [2]. CYP76AD1 is one of the two precursors of betacyanin, which catalyzes the conversion of L-DOPA to cylco-DOPA [9,18]. Silencing of the *CYP76AD1* gene results in the production of only yellow betaxanthins and the loss of red pigment. Introduction of *CYP76AD1* into the yellow betalain mutants (an insertion in CYP76AD1 maps to the R locus that is responsible for yellow versus red pigmentation) produces both yellow and red pigmentation [9]. The expression of CYP76AD1 in yeast confirms its role in betalain biosynthesis [9,11]. Moreover, heterologous expression of *BvCYP76AD1* or *CqCYP76AD1* in tobacco results in an accumulation of betalains [11]. In our previous study, *comp16058_c0_seq1* (named *HmoCYP76AD1*), an important candidate gene of *cyt-P450* genes involved in the betalain biosynthetic pathway, was identified from pitaya by RNA-Seq and its amino acid sequence was 74% homologous with *BvCYP76AD1* [10]. In this study, the expression and promoter of *HmoCYP76AD1* were analyzed. Expression levels of *HmoCYP76AD1* increased gradually during pulp coloration and decreased at the full maturation stage (Figure 3). A typical W-box motif with a core sequence of (C/T)TGAC(C/T) was found in the *HmoCYP76AD1* promoters (Table 1). These results indicate that *HmoCYP76AD1* is possibly involved in the betalain biosynthesis of pitaya.

The WRKY proteins are plant-specific proteins involved in multiple biological processes in plants. WRKYs not only play important roles in biotic and abiotic stresses in many plant processes [19,20,21], but are also involved in the regulation of plant growth and developmental processes including trichome [22] and seed development [23], germination [24], leaf senescence [25] and the synthesis of secondary metabolites including tryptamine [26], alkaloids [27], terpenes [28] and betalains [16]. WRKY proteins are clustered into three major groups (I, II and III) and group II can be further subdivided into five subgroups (IIa, IIb, IIc, IId and IIe). WRKY proteins can activate or inhibit the expression of downstream genes by binding the W-box ((C/T)TGAC(C/T)) elements of its target gene promoter [16,29,30]. In our previous study, a HmoWRKY44 TF belonging to a member of the Group I; WRKY family was obtained from pitaya fruit. The W-box motif of HmoWRKY44 is predicted to be from 581 bp downstream (+) to 1351 bp upstream (−) of the transcription start site [16]. HmoWRKY44 could activate the HmoCYP76AD1 expression by binding to its promoter, which is responsible for the betalain biosynthesis of pitaya [16]. In the present work, HmoWRKY40, a novel WRKY TF, was obtained from pitaya transcriptome data. The HmoWRKY40 had one WRKY domain at 120 bp (−) upstream and belonged to the Group IIa WRKY family (Figure 2). Expression levels of *HmoWRKY40* increased significantly during the coloration period (23rd to 25th DAAP) of the Guanhuahong pitaya pulps (Figure 3), which was consistent with the expression trend of *HmoCYP76AD1* and the betalain accumulation of the pulp (Figure 1). These results suggest that HmoWRKY40 is involved in the betalain biosynthesis of pitaya.

The WRKY proteins are typically nuclear-localized proteins [30,31,32,33]. In our study, HmoWRKY40 was located in the nucleus (Figure 4). Silencing of the HmoWRKY40 resulted in a decrease in betalain contents in the pitaya scale (Figure 6). Yeast single-hybrid and dual luciferase experiments showed that HmoWRKY40 can activate the expression of the *HmoCYP76AD1* gene by binding to its promoter, which participates in the regulation of betalain biosynthesis (Figure 7 and Figure 8). The LUC/REN ratio of CaMV35S-HmoWRKY40 co-transduced with CaMV35S-REN/HmoCYP76AD1-pro had a relatively higher expression than that of the control (Figure 8), which is consistent with HmoWRKY44 [16]. These results indicate that HmoWRKY40 plays a key role in pitaya betalain biosynthesis through regulating the expression of the *HmoCYP76AD1* gene.

In summary, a novel WRKY transcription factor HmoWRKY40, belonging to a member of the Group IIa WRKY family, was obtained from pitaya. HmoWRKY40 activated the *HmoCYP76AD1* expression by binding to its promoter. Silencing of HmoWRKY40 resulted in a significant reduction in betalain contents. These results suggest that HmoWRKY40 plays a key role in the betalain biosynthesis of pitaya. The present study provides new insights into the transcriptional regulation of genes related to betalain biosynthesis in pitaya.

## 4. Materials and Methods

### 4.1. Materials

Two pitaya cultivars, Guanhuahong (red peel and green scales with red pulp, *H. monacanthus*) and Hongguan No. 1 (red peel and red scales with red pulp, *H. monacanthus*) from the same orchard in Jinsuinong (Zhongluotan Village, Guangzhou City, Guangdong Province, China), were used as materials. Fruits of Guanhuahong pitayas were collected on the 19th, 23rd, 25th, 27th and 29th day after artificial pollination (DAAP) (Figure 1A). Scales of the Hongguan No. 1 pitaya on the 23rd DAAP were used for virus-induced gene silencing (VIGS). All samples were frozen in liquid nitrogen immediately and stored at −80 °C for future analysis.

### 4.2. Measurement of Betalain Contents

Freeze-dried pitaya pulps were ground into powder in liquid nitrogen. Betalain contents were extracted and measured following the procedure of Hua et al. (2016) [10]. All measurements were repeated three times. A two-tailed t-test was used to determine the significance at *p* < 0.05 and *p* < 0.01 using the SPSS 17.0 software.

### 4.3. Gene Cloning and Sequence Analyses

Total RNA was extracted using the EASYspin Plus polysaccharide polyphenol complex plant RNA rapid extraction kit (RN53) (Aidlab, Beijing, China). The RNA quality and concentration were assessed respectively by 1.2% agarose gel and spectrophotometry. RNA samples with an OD_260_/OD_280_ ratio of 1.9–2.1 were reverse-transcribed into cDNA using the PrimeScript^TM^ RT reagent Kit with the gDNA Eraser (Perfect Real Time) according to the manufacturer’s instructions (TaKaRa, Shiga, Japan).

HmoWRKY40 was obtained from the RNA-Seq database [10]. The open reading frame (ORF) of the HmoWRKY40 sequence was cloned by gene-specific primer pairs (Supplementary Table S1) and the NCBI database (http://www.ncbi.nlm.nih.gov/) was used to find similar sequences for homology alignment. Sequences were aligned using the ClustalX and GeneDoc software. The phylogenetic tree was constructed using the Maximum likelihood method in MEGA7.0. The theoretical isoelectric point (pI) and relative molecular mass of HmoWRKY40 were predicted at http://web.expasy.org/compute_pi/.

### 4.4. RT-qPCR Analyses

RT-qPCR was performed on the Applied Biosystems 7500 Real-Time PCR System (Applied Biosystems, CA, USA) and the reaction system was run according to the instructions in the ChamQ^TM^ SYBR qPCR Master MIXEx (Planta) kit (Vazyme, Nanjing, China). A total of 20.0 μL reaction system included 10.0 μL of ChamQ SYBR qPCR Master Mix (2×), 0.4 μL of each forward and reverse primer (10 μM), 1.0 μL cDNA (70 ng) and 8.2 μL ddH_2_O. The samples were initially denaturated at 94 ℃ for 5 min before being subjected to 30 cycles consisting of 15 s at 95 °C (denaturation), 30 s at 56 °C (annealing) and 40 s at 72 °C (extension). The relative expression levels of HmoWRKY40 were calculated by the formula 2^−∆∆C^T [34]. Primers are listed in the Supplementary Table S1.

### 4.5. Subcellular Localization of HmoWRKY40

The full-length coding sequence of *HmoWRKY40* was inserted into the pGreen-35S-GFP. Cells of the *Agrobacterium tumefaciens* strain of GV3101-pSoup, carrying 35S-HmoWRKY40-GFP and the pGreen-35S-GFP positive control, were separately co-expressed with At1g22590-RFP (for nuclear positioning) in a ratio of 1:1 to infect tobacco (*Nicotiana benthamiana*) leaves [35]. Transient expression of GFP and RFP signals were observed using a laser confocal microscope (Zeiss Axioskop 2 Plus, Germany) after two days of infiltration.

### 4.6. Gene Silencing of HmoWRKY40

The full length of the *HmoWRKY40* fragment was ligated to the pTRV2 vector; pTRV1, pTRV2 and pTRV2-HmoWRKY40 were transformed into the *Agrobacterium tumefaciens* strain GV3101 (primers are listed in Supplementary Table S1). The bacterial cells were resuspended to an OD_600_ of 0.4 using an MMA buffer (10 mM MES, 10 mM MgCl_2_, 100 μM acetosyringone) according to the proportion of 1:1. VIGS was performed as described in our previous study [13].

### 4.7. Promoter Analysis of HmoCYP76AD1

Total genomic DNA was extracted using a CTAB genomic DNA Extraction Kit (DN14) (Aidlab, China) and RNA was removed with Ribonuclease A (RNase A) (TaKaRa, Shiga, Japan). *HmoCYP76AD1* was cloned using specific primer pairs (Supplementary Table S1). Promoter elements and the conserved binding domain of *HmoCYP76AD1* were analyzed by the Plant-CARE website (http://bioinformatics.psb.ugent.be/webtools/plantcare/html/).

### 4.8. Yeast One-Hybrid Experiment

The promoter of *HmoCYP76AD1* was ligated to the pAbAi yeast reporter vector to form a pPromoter-AbAi recombinant bait vector. The HmoWRKY40 was ligated into the expression vector pGADT7 to construct the pGADT7-AD recombinant vector and the pGADT7-HmoWRKY40 vector plasmid was used to transform the positively verified pHmoCYP76AD1-AbAi (primers are listed in Supplementary Table S1). The bait yeast strain was cultured at 30 °C until colonies appeared (3–5 d). The interaction between TF and the promoter was judged by the yeast growth [36].

### 4.9. Transcriptional Activity

The ORFs of the HmoWRKY40 were ligated into the vector pGBKT7; pGBKT7-p53 and pGADT7-T were co-transduced as the positive controls and the corresponding bait with an empty pGBKT7 vector was used as the negative control. The vector plasmid pGBKT7-HmoWRKY40 was transformed into the Y2HGold yeast competent cells. The yeast strain was grown on the SD/-Trp plate and cultured at 30 °C until single individual colonies appeared. The single colony was picked up and suspended in 0.9% NaCl solution. Then, suspended cells were coated on plates containing SD/-Trp-His-Ade/Kan and cultured at 30 °C for 3–5 d. X-α-gal (20 μg/mL) was added to a final concentration of 20 μg/mL on the SD/-Trp-His-Ade/Kan plate. The transcriptional activation ability of HmoWRKY40 was investigated according to the yeast color.

### 4.10. Dual-Luciferase Reporter Assay

The full length of HmoWRKY40 was ligated into the pEAQ vector as an effector. The promoter sequence of *HmoCYP76AD1* was ligated to the pGReenII 0800-LUC (LUC0800) vector to construct the corresponding recombinant dual luciferase reporter vector. The pHmoCYP76AD1-pro-LUC was transformed into the *A. tumefaciens* strain GV3101. The effector and reporter bacteria cells were resuspended according to the proportion of 9:1 and used for injecting tobacco (*N. benthamiana*) leaves. Two days after infiltration, leaves were determined with a Luminoskan Ascent Microplate Luminometer (Thermo Scientific, Rockford, IL, USA) according to the method in the dual luciferase assay kits (Promega, Madison, WI, USA) [37].

## Figures and Tables

**Table 1:** Motif analyses of the *HmoCYP76AD1* promoter.

Site Names	Positions	Sequences	Functions
AAGAA-motif	353 (+)	GAAAGAA	
ABRE	162 (−); 771 (−)	ACGTG/CGTACGTGCA	Cis-acting element involved in the abscisic acid responsiveness
ABRE3a	162 (−)	TACGTG	
ABRE4	162 (+)	CACGTA	
ARE	659 (−)	AAACCA	Cis-acting regulatory element essential for the anaerobic induction
AT~TATA-box	310 (+)	TATATA	
Box III	628 (+)	ATCATTTTCACT	Protein binding site
CAAT-box	11 (+); 60 (−); 179372 (−); 191 (−); 203 (+); 228 (+); 372 (+); 378 (+); 403 (−); 451 (−); 512 (−); 527 (−); 640 (−); 645 (+); 746 (+); 781 (+); 782 (+)	CCAAT/CAAAT	Common cis-acting element in promoter and enhancer regions
G-box	162 (−);	TACGTG	Cis-acting regulatory element involved in light responsiveness
GARE-motif	591 (+);	TCTGTTG	Gibberellin-responsive element
GCN4_motif	570 (−);	TGAGTCA	Cis-regulatory element involved in endosperm expression
MRE	538 (+);543 (+);	AACCTAA	MYB binding site involved in light responsiveness
MYB-binding site	471 (+); 592 (−)	TAACCA/CAACAG	
MYC	190 (+); 718 (−); 720 (+); 746 (−)	CATTTG/CATGTG	
MYB	566 (+)	TAACTG	
STRE	873 (−)	AGGGG	stress response element
TATA	550 (−)	TATAAAAT	
TATA-box	135 (−); 136 (+); 166 (+); 174 (−); 194 (−); 196 (+); 213 (−); 245 (+); 246 (−); 247 (+); 262 (+); 263 (−)264 (+); 309 (+); 310 (+); 312 (+); 343 (+); 551 (−); 552 (−); 553 (−); 554 (−); 728 (−); 729 (−); 730 (−)	TATAA/TATA/TATAAA/TATAAAA/TATATA/ATATAT/ATTATA/TACAAAA/TATACA/CCTATAAAAA/TATTTAAA	Core promoter element around -30 of transcription start
WRE3	487 (+)	CCACCT	Wnt-responsive element
W-box	120 (−)	TTGACT	WRKY binding site

## Data Availability

Data is contained within the article and supplementary material.

## Supplementary Materials

The following are available online at https://www.mdpi.com/1422-0067/22/4/2171/s1.

## Abbreviations

cDNA	Complementary DNA
CTAB	Hexadecyltrimethylammonium bromide
CYP	Cytochrome P450
DAAP	Day after artificial pollination
DOD	Dioxygenase
DOPA	4,5-dihydroxy-phenylalanine
GFP	Green fluorescent protein
GTs	Glucosyltransferases
L-DOPA	L-3,4-dihydroxyphenylalanine
MES	(2-Nmorpholino) ethanesulfonic acid
MMA	MES+MgCl_2_+acetosyringone
MYB	Myb proto-oncogene protein
NCBI	National Center for Biotechnology Information
ORF	Open reading frame
pI	Theoretical isoelectric point
RFP	Red fluorescent protein
RNA-Seq	RNA sequencing
RT-qPCR	Reverse transcription quantitative real-time polymerase chain reaction
TFs	Transcription factors
TYR	Tyrosinase
VIGS	Virus-induced gene silencing

## References

[1] Fernando G.H., Francisco G.C. (2012). Characterization of recombinant *Beta vulgaris* 4,5-DOPA-extradiol-dioxygenase active in the biosynthesis of betalains. Planta.

[2] Stafford H.A. (1994). Anthocyanins and betalains: Evolution of the mutually exclusive pathways. Plant Sci..

[3] Jain G., Gould K.S. (2015). Functional significance of betalain biosynthesis in leaves of *Disphyma australe* under salinity stress. Environ. Exp. Bot..

[4] Jain G., Schwinn K.E., Gould K.S. (2015). Betalain induction by l-DOPA application confers photoprotection to saline-exposed leaves of *Disphyma australe*. New Phytol..

[5] Lakhotia P., Singh K.P., Singh S.K., Singh M.C., Swaroop K. (2014). Influence of biotic and abiotic elicitors on production of betalain pigments in bougainvillea callus cultures. Indian J. Hortic..

[6] Gengatharan A., Dykes G.A., Choo W.S. (2015). Betalains: Natural plant pigments with potential application in functional foods. LWT Food Sci. Technol..

[7] Clifford T., Howatson G., West D.J., Stevenson E.J. (2015). The potential benefits of red beetroot supplementation in health and disease. Nutrients.

[8] Da Silva D.V.T., dos Santos Baião D., de Oliveira Silva F., Alves G., Perrone D., Del Aguila E.M., Paschoalin V.M.F. (2019). Betanin, a natural food additive: Stability, bioavailability, antioxidant and preservative ability assessments. Molecules.

[9] Hatlestad G.J., Sunnadeniya R.M., Akhavan N.A., Gonzalez A., Goldman I.L., McGrath J.M., Lloyd A.M. (2012). The beet R locus encodes a new cytochrome P450 required for red betalain production. Nat. Genet..

[10] Hua Q.Z., Chen C.J., Chen Z., Chen P.K., Ma Y.W., Wu J.Y., Zheng J., Hu G.B., Zhao J.T., Qin Y.H. (2016). Transcriptomic analysis reveals key genes related to betalain biosynthesis in pulp coloration of *Hylocereus polyrhizus*. Front. Plant Sci..

[11] Polturak G., Breitel D., Grossman N., Sarrion-Perdigones A., Weithorn E., Pliner M., Orzaez D., Granell A., Rogachev I., Aharoni A. (2016). Elucidation of the first committed step in betalain biosynthesis enables the heterologous engineering of betalain pigments in plants. New Phytol..

[12] Polturak G., Aharoni A. (2018). “La Vie en Rose”: Biosynthesis, sources, and applications of betalain pigments. Mol. Plant.

[13] Xie F.F., Hua Q.Z., Chen C.B., Zhang L.L., Zhang Z.K., Chen J.Y., Zhang R., Zhao J.S., Hu G.B., Zhao J.T. (2020). Transcriptomics-based identification and characterization of *glucosyltransferases* involved in betalain biosynthesis in *Hylocereus megalanthus*. Plant Physiol. Bioch..

[14] Stracke R., Holtgräwe D., Schneider J., Pucker B., Sörensen T.R., Weisshaar B. (2014). Genome-wide identification and characterisation of R2R3-MYB, genes in sugar beet (*Beta vulgaris*). BMC Plant Biol..

[15] Hatlestad G.J., Akhavan N.A., Sunnadeniya R.M., Elam L., Cargile S., Hembd A., Gonzalez A., McGrath J.M., Lloyd A.M. (2014). The beet Y locus encodes an anthocyanin MYB-like protein that activates the betalain red pigment pathway. Nat. Genet..

[16] Cheng M.N., Huang Z.J., Hua Q.Z., Shan W., Kuang J.F., Lu W.J., Qin Y.H., Chen J.Y. (2017). The WRKY transcription factor HpWRKY44 regulates *CytP450-like1* expression in red pitaya fruit (*Hylocereus polyrhizus*). Hortic. Res..

[17] Ibrahim S.R.M., Mohamed G.A., Khedr A.I.M., Zayed M.F., EI-Kholy A.A.E.S. (2018). Genus *Hylocereus*: Beneficial phytochemicals, nutritional importance, and biological relevance-A review. J. Food Biochem..

[18] Sunnadeniya R., Bean A., Brown M., Akhavan N., Hatlestad G., Gonzalez A., Symonds V.V., Lloyd A. (2016). Tyrosine hydroxylation in betalain pigment biosynthesis is performed by cytochrome P450 enzymes in beets (*Beta vulgaris*). PLoS ONE.

[19] Chen L.G., Song Y., Li S.J., Zhang L.P., Zou C.S., Yu D.Q. (2012). The role of WRKY transcription factors in plant abiotic stresses. BBA.

[20] Zhang Y., Feng J.C. (2014). Identification and characterization of the grape WRKY family. Biomed Res. Int..

[21] Joshi R., Wani S.H., Singh B., Bohra A., Dar Z.A., Lone A.A., Pareek A., Singla-Pareek S.L. (2016). Transcription factors and plants response to drought stress: Current understanding and future directions. Front. Plant Sci..

[22] Johnson C.S., Kolevski B., Smyth D.R. (2002). *TRANSPARENT TESTA GLABRA2*, a trichome and seed coat development gene of Arabidopsis, encodes a WRKY transcription factor. Plant Cell.

[23] Luo M., Dennis E.S., Berger F., Peacock W.J., Chaudhury A. (2005). *MINISEED3* (*MINI3*), a WRKY family gene, and *HAIKU2* (*IKU2*), a leucine-rich repeat (*LRR*) *KINASE* gene, are regulators of seed size in Arabidopsis. Proc. Natl. Acad. Sci. USA.

[24] Jiang W.B., Yu D.Q. (2009). Arabidopsis WRKY2 transcription factor mediates seed germination and postgermination arrest of development by abscisic acid. BMC Plant Biol..

[25] Miao Y., Laun T., Zimmermann P., Zentgraf U. (2004). Targets of the WRKY53 transcription factor and its role during leaf senescence in Arabidopsis. Plant Mol. Biol..

[26] Jadaun J.S., Kushwaha A.K., Sangwan N.S., Narnoliya L.K., Mishra S., Sangwan R.S. (2020). WRKY1-mediated regulation of tryptophan decarboxylase in tryptamine generation for withanamide production in *Withania somnifera* (Ashwagandha). Plant Cell Rep..

[27] Schluttenhofer C., Yuan L. (2015). Regulation of specialized metabolism by WRKY transcription factors. Plant Physiol..

[28] Singh A.K., Kumar S.R., Dwivedi V., Rai A., Pal S., Shasany A.K., Nagegowda D.A. (2017). A WRKY transcription factor from *Withania somnifera* regulates triterpenoid withanolide accumulation and biotic stress tolerance through modulation of phytosterol and defense pathways. New Phytol..

[29] Xie Z., Zhang Z.L., Zou X.L., Huang J., Ruas P., Thompson D., Shen Q.X.J. (2005). Annotations and functional analyses of the rice *WRKY* gene superfamily reveal positive and negative regulators of abscisic acid signaling in aleurone cells. Plant Physiol..

[30] Jiang J.J., Ma S.H., Ye N.H., Jiang M., Cao J.S., Zhang J.H. (2017). WRKY transcription factors in plant responses to stresses. J. Integr. Plant Biol..

[31] Gong X.Q., Zhang J.Y., Hu J.B., Wang W., Wu H., Zhang Q.H., Liu J.H. (2015). FcWRKY70, a WRKY protein of *Fortunella crassifolia*, functions in drought tolerance and modulates putrescine synthesis by regulating *arginine decarboxylase* gene. Plant Cell Environ..

[32] Phukan U.J., Jeena G.S., Shukla R.K. (2016). WRKY transcription factors: Molecular regulation and stress responses in plants. Front. Plant Sci..

[33] Ye Y.J., Xiao Y.Y., Han Y.C., Shan W., Fan Z.Q., Xu Q.G., Kuang J.F., Lu W.J., Lakshmanan P., Chen J.Y. (2016). Banana fruit VQ motif-containing protein5 represses cold-responsive transcription factor MaWRKY26 involved in the regulation of JA biosynthetic genes. Sci. Rep..

[34] Livak K.J., Schmittgen T.D. (2001). Analysis of relative gene expression data using real-time quantitative PCR and the 2^−∆∆C^T Method. Methods.

[35] Lee L.Y.C., Hou X.L., Fang L., Fan S.G., Kumar P.P., Yu H. (2012). *STUNTED* mediates the control of cell proliferation by GA in *Arabidopsis*. Development.

[36] Ouwerkerk P.B., Meijer A.H. (2001). Yeast one-hybrid screening for DNA-protein interactions. Curr. Protoc. Mol. Biol..

[37] Hellens R.P., Allan A.C., Friel E.N., Bolitho K., Grafton K., Templeton M.D., Karunairetnam S., Gleave A.P., Laing W.A. (2005). Transient expression vectors for functional genomics, quantification of promoter activity and RNA silencing in plants. Plant Methods.

