# Supplementary material for: A Novel WRKY Transcription Factor HmoWRKY40 Associated with Betalain Biosynthesis in Pitaya (Hylocereus monacanthus) through Regulating HmoCYP76AD1
Source: Int J Mol Sci. 2021 Feb 22;22(4):2171. doi: 10.3390/ijms22042171 (PMC7926660; doi:10.3390/ijms22042171)
Supplement: Supplementary file 1 [file ijms-22-02171-s001.zip › Supplementary files/Supplementary Table S1.docx]

**Supplementary Table S1. Primers used in this study**

| **Purpose** | **Gene names** | **Primer sequences** |
| --- | --- | --- |
| Full length cloning | HmoWRKY40 | ATGGATTATTCAACGTGGTTAAGCTCAGC |
|  |  | ATCAGTTGAGGTTTGTGCAGATCTCC |
| Promoter cloning | HmoCYP76AD1 | GACATAAGACTCAATTTACTTGGAA |
|  |  | AGTTATGAAGTAAAAGATTGAGGCA |
| RT-qPCR | HmoWRKY40 | GTAATGAGTTGGTGGGCAAT |
|  |  | AGCCTCTTCCTTTGGTTTCT |
|  | HmoCYP76AD1 | GCTCCAGCCGAACCATACCC |
|  |  | TCTTCCTAAAACTCCGCCAT |
| Subcellular  localization | HmoWRKY40 | GGTATCGATAAGCTTATGGATTATTCAACG |
|  |  | CATACTAGTGGATCCATCAGTTGAGGTTTG |
| Trans-activation  activity assay | pGBKT7-HmoWRKY40 | catatggccatggaggccgaattcATGGATTATTCAACGTGGTTAAGCTCAGC |
|  |  | ggccgctgcaggtcgacggatcc TTAATCAGTTGAGGTTTGTGCAGATCTCC |
| Yeast one-hybrid | pGADT7-HmoWRKY40 | CAGTGAATTCCACCCGGGATGGATTATTCAACGTGGTTAAGCTCAGC |
|  |  | TATCGATGCCCACCCGGGATCAGTTGAGGTTTGTGCAGATCTCC |
|  | pAbAi- HmoCYP76AD1 | AAAAGCTTGAATTCGAGCTCGACATAAGACTCAATTTACTTGGAA |
|  |  | GAGCACATGCCTCGAGAGTTATGAAGTAAAAGATTGAGGCA |
| Dual LUC  assay | PEAQ-HmoWRKY40 | CAAATTCGCGACCGGTATGGATTATTCAACGTGG |
|  |  | AGTTAAAGGCCTCGAGTTAATCAGTTGAGGTTTG |
|  | LUC- HmoCYP76AD1 | CTATAGGGCGAATTGGGTACCGACATAAGACTCAATTTACTTGGAA |
|  |  | CGCTCTAGAACTAGTGGATCCTTATGAAGTAAAAGATTGAGGCA |
| VIGS | pTRV2-HmoWRKY40 | gcctccatggggatcATGGATTATTCAACGTGGTTAAGCT |
|  |  | cttcgggacatgcccTTAATCAGTTGAGGTTTG |

**Supplementary Text S1.** Nucleotide sequence of *HmoCYP76AD1* promoter. W-box (T/C)TGAC(T/C) is indicated in box. Translation start site (ATG) is shown in red.

*>*HmoCYP76AD1

GACATAAGACTCAATTTACTTGGAAAAACGTTTTCCACAGAAAATGATTTCTTGGAAAAAATTGTTTTCCGCAAAAAATGATTTTTCGAGAAAACAACTTCCTTTGGAACAAAACACTATCTTAATTAAAACATCTTATATGTGCTTTGGAAGGACACGCACCACGTATATGATTTTTTATTGGACATTTCATTTGTATATTTCAATAAACTTTTTTGTAGACAACATCAATCTCTTTGTCCTTAATTATAAACCCTTAAAAATTATATTTCACATCTTTTCATTATGTCAACAAAAAAATCCTATGAGATATATAGCATTTTGCACTGAATAAAAATGACCATATTTAAATGGAAAGAAAAAAATCATGTACAATTTCAAATTTCAGCATTTTTTTTTAATAATTGAAGCCAAAGGCGGGCCATGGCAGGAGCAGTAAAACAGATAACTTATTTGGCCGGCACTTGCAAGTAACCAAAGGATCGCACCACCTCCGCTGGTCTGTAAATAATATTGTCCAGCCTAAAATTGATCCATCAACCTAACCTAAATTTTATAGAATAGAATAACTGACTCAGCTCAAGAGATCGGTCTGTTGTCAAACCTCAGCCTCACTAGCTCATTCAAAATAATTTTCACTATTGCCAAATCTCCTCTTATGGTTCTCCAAACATTATGCACACCCATGCCTAGCTGGATTCCCTTTCCTACCTACATGCACATGTGTGTTTATAAATGTATGTCTGCAAATGTTCGCTTATCA**AGTCAA**GGTGAACGTACGCCAATAAGCCTGCATGTATTACATGCATACGCATTCCTAGCTAGCTAAAATTCCCTGCATTCTCTCTCTCCTTCTCTCATCCTCCCCCCCCCCCCCTTCCCAAAAGCATG

**Supplementary Text S2.** cDNA sequences of *HmoWRKY40* and *HmoCYP76AD1*.

>*HmoWRKY40*

ATGGATTATTCAACGTGGTTAAGCTCAGCTTCATTGGATCTGAGTCGTAGCAATGATCACAATCATGGCAATGACAATACGCTCCGGCTTTTCGATGATTCTCCTGTTCGGGATGGTCTTAAGACTGCCATCAAAGTTGAGACGATCCAAACCTGCAGCTCCAATGAAGTTGATCGCCAACCTTTGATCAAAGATGAGGCAGGAGTTCTGATGGAAGAGTTGAAGAAGGCAAACGAAGAGAACAGGAGGTTAACCGAAATGTTAACGGTCGTATGTGACAATTACAATGATTTGAAGAGGCAGTTGGCGGATTACATGACTAAAGCTGCAAGTAATGAGTTGGTGGGCAATGTGGCCAAGAAAAGAAAGATCGAGAATTCCACCAATAGTAATAACAACAACAATAACAACAACAGTAACAAGATTATTGGCAGTAATAATGTCGACAGTGGTTCGAGCGATGAAGAAGATTCGTGTGAGAAACCAAAGGAAGAGGCTATTAAAGCCAAGGTCACCAGAGTTGCTGTTCGAACTGAAGCTTCTGACTCCACCCTTATCCTGAAGGATGGATATCAATGGAGGAAATACGGGCAAAAGGTGACTAGGGATAACCCTTGCCCTAGAGCTTACTTCAAGTGCTCCTTCGCGCCTAGTTGTCCGGTTAAAAAGAAGGTTCAAAGAAGTCTAGAAGACCAATCAATGCTAGTTGCAACCTACGAAGGGGAGCACAACCATGCACCTCCATCCCAACAGGAGCCAGCACTGGGCCCAAACCGGTCCTTCAGCCTCGGCTCCGTCGCATGTAACGCGACCCTAGCCTCATCCGGACCCACAGTCACTCTTGACCTAACCAAGCCCAAGCCTAGCACTGCTAAAAATGACACAACTCCAAAAACATTAAGCAGCAATAACGTCAGTTCTGCCCAACTGTTGAAATTCAACTCCCCTGAATTTCAGAAGCTTTTGGCAGAACAAATGGCTTCTTCTTTGACAAAAGATCCCAACTTCACAGCTGCTCTTGCTGCTGCCATTTCTGGGAGATCTGCACAAACCTCAACTGATTAA

> *HmoCYP76AD1*

ATGGATAGCCCAACCCTCTCGCTTTTCATCTTTGCCTCAATCTTTTACTTCATAACTTTTCAAATTGTGAAGCTAGGGTTTAATGTGGTCATGACCTCTAAAAAAACCAAAAGAAGAAGACCCCCCTTACCTCCGGGCCCCAAGCCATTGCCCATCATAGGCAATGTGCTCGAGCTCGGGCCGAAGCCACACCGCTCGTTCGCCGACCTAGCCAAGGTCCATGGTCCACTCATGTCCCTCCGGCTAGGTAGTGTGACCACGATTATCGTGTCATCCTCTGATGTTGCCAAAGAAATGTTCCTTAAAAATGACCAACCCTTGAGCTCCAGCCGAACCATACCCAACTCAGTCACGGCTGGGGATCACCACATGCTGACCATGTCTTGGCTCCCAGTCTCCCCCAAATGGCGGAGTTTTAGGAAGATCACCACCTTCCACCTTCTCTCCCCCCAGCGCCTCGATGCTTGCTCTAGCCTTAGGCAAGCCAAGGTGCAGCAGCTATTCGAGTACGTTCTGGAATGTTCTAGAACCGGCCAGGCCGTCGATATAGGCAAGGCTGCTTTCACGACGTCCCTTAACTTGTTGTCCAAGCTGTTTTTTTCTTTAGAGTTGGCTCACCATAGATCTAGCAAGTCTCAAGAGTTTAAGGACTTAATTTGGGATATTATGGAGGATATTGGGAAGCCTAATTACGCGGATTATTTCCCATGCTTAAAGTACTTTGACCCATGTGGAATACGACGTCGTTTGGCAAATAGTTTTGAGAAATTAATTGAGGTCTTTCAAGGTATTATTCGTCAAAGGCTATCCCTGTCATCTGGCTCTCATACTCATAATGATGTGTTAGATGTTCTTCTTCAATTGTACAACCAAGAGGAACTCACCATGGACGAGATAAACCATCTGCTCGTGGATATATTTGATGCCGGAACAGACACCACTTCCAGTACATTTGAATGGGCCATGGCTGAGTTAATTAAAAATCCGACGATGATGGAGAAAGCTCAAGCTGAAATCAAAGTGGTTCTTGGGAAACAGTCGCATATTCAAGAGTCCGATATCCCAAAATTGCCTTATTTGCGGGCAATTATCAAAGAAACATTGCGTCTACACCCTCCTACTGTATTCCTCCTACCTCGTAAGGCTGAGACCGATGTGGAACTCTATGGCTACACCGTACCAAAAAATGCACAAATACTGGTGAACTTGTGGGCCTTAGGTCGAGACCCCAAAGTTTGGGAAAACCCAGAGGTGTTCTTACCTGAAAGGTTCCTGACTTGCGACATCGATGTTAAAGGAAGAGATTTTGGACTACTGCCTTTTGGGGCAGGAAGGCGAATATGTCCTGGGATGAATTTGGCGTACAGAATGCTGACCTTAATGCTCGCTACGCTTCTACAATCGTTTGATTGGAAACTCCCAAATGAGATGAACTCCAAGAATTTGGACATGGATGAAAAGTTTGGAATAGCATTGCAAAAGACTAAACCCCTTGAAATTATTCCCGTTTGCAAGGATTGA
