# Supplementary material for: A Novel WRKY Transcription Factor HmoWRKY40 Associated with Betalain Biosynthesis in Pitaya (Hylocereus monacanthus) through Regulating HmoCYP76AD1
Source: Int J Mol Sci. 2021 Feb 22;22(4):2171. doi: 10.3390/ijms22042171 (PMC7926660; doi:10.3390/ijms22042171)
Supplement: Supplementary file 1 [file ijms-22-02171-s001.zip › Supplementary files/Supplementary Text S1.docx]

**Supplementary Text S1.** Nucleotide sequence of *HmoCYP76AD1* promoter. W-box (T/C)TGAC(T/C) is indicated in box.

*>*HmoCYP76AD1

GACATAAGACTCAATTTACTTGGAAAAACGTTTTCCACAGAAAATGATTTCTTGGAAAAAATTGTTTTCCGCAAAAAATGATTTTTCGAGAAAACAACTTCCTTTGGAACAAAACACTATCTTAATTAAAACATCTTATATGTGCTTTGGAAGGACACGCACCACGTATATGATTTTTTATTGGACATTTCATTTGTATATTTCAATAAACTTTTTTGTAGACAACATCAATCTCTTTGTCCTTAATTATAAACCCTTAAAAATTATATTTCACATCTTTTCATTATGTCAACAAAAAAATCCTATGAGATATATAGCATTTTGCACTGAATAAAAATGACCATATTTAAATGGAAAGAAAAAAATCATGTACAATTTCAAATTTCAGCATTTTTTTTTAATAATTGAAGCCAAAGGCGGGCCATGGCAGGAGCAGTAAAACAGATAACTTATTTGGCCGGCACTTGCAAGTAACCAAAGGATCGCACCACCTCCGCTGGTCTGTAAATAATATTGTCCAGCCTAAAATTGATCCATCAACCTAACCTAAATTTTATAGAATAGAATAACTGACTCAGCTCAAGAGATCGGTCTGTTGTCAAACCTCAGCCTCACTAGCTCATTCAAAATAATTTTCACTATTGCCAAATCTCCTCTTATGGTTCTCCAAACATTATGCACACCCATGCCTAGCTGGATTCCCTTTCCTACCTACATGCACATGTGTGTTTATAAATGTATGTCTGCAAATGTTCGCTTATCA**AGTCAA**GGTGAACGTACGCCAATAAGCCTGCATGTATTACATGCATACGCATTCCTAGCTAGCTAAAATTCCCTGCATTCTCTCTCTCCTTCTCTCATCCTCCCCCCCCCCCCCTTCCCAAAAGC
