# Supplementary material for: A Novel WRKY Transcription Factor HmoWRKY40 Associated with Betalain Biosynthesis in Pitaya (Hylocereus monacanthus) through Regulating HmoCYP76AD1
Source: Int J Mol Sci. 2021 Feb 22;22(4):2171. doi: 10.3390/ijms22042171 (PMC7926660; doi:10.3390/ijms22042171)
Supplement: Supplementary file 1 [file ijms-22-02171-s001.zip › Supplementary files/Supplementary Text S2.docx]

**Supplementary Text S2.** cDNA sequences of *HmoWRKY40* and *HmoCYP76AD1*.

>*HmoWRKY40*

ATGGATTATTCAACGTGGTTAAGCTCAGCTTCATTGGATCTGAGTCGTAGCAATGATCACAATCATGGCAATGACAATACGCTCCGGCTTTTCGATGATTCTCCTGTTCGGGATGGTCTTAAGACTGCCATCAAAGTTGAGACGATCCAAACCTGCAGCTCCAATGAAGTTGATCGCCAACCTTTGATCAAAGATGAGGCAGGAGTTCTGATGGAAGAGTTGAAGAAGGCAAACGAAGAGAACAGGAGGTTAACCGAAATGTTAACGGTCGTATGTGACAATTACAATGATTTGAAGAGGCAGTTGGCGGATTACATGACTAAAGCTGCAAGTAATGAGTTGGTGGGCAATGTGGCCAAGAAAAGAAAGATCGAGAATTCCACCAATAGTAATAACAACAACAATAACAACAACAGTAACAAGATTATTGGCAGTAATAATGTCGACAGTGGTTCGAGCGATGAAGAAGATTCGTGTGAGAAACCAAAGGAAGAGGCTATTAAAGCCAAGGTCACCAGAGTTGCTGTTCGAACTGAAGCTTCTGACTCCACCCTTATCCTGAAGGATGGATATCAATGGAGGAAATACGGGCAAAAGGTGACTAGGGATAACCCTTGCCCTAGAGCTTACTTCAAGTGCTCCTTCGCGCCTAGTTGTCCGGTTAAAAAGAAGGTTCAAAGAAGTCTAGAAGACCAATCAATGCTAGTTGCAACCTACGAAGGGGAGCACAACCATGCACCTCCATCCCAACAGGAGCCAGCACTGGGCCCAAACCGGTCCTTCAGCCTCGGCTCCGTCGCATGTAACGCGACCCTAGCCTCATCCGGACCCACAGTCACTCTTGACCTAACCAAGCCCAAGCCTAGCACTGCTAAAAATGACACAACTCCAAAAACATTAAGCAGCAATAACGTCAGTTCTGCCCAACTGTTGAAATTCAACTCCCCTGAATTTCAGAAGCTTTTGGCAGAACAAATGGCTTCTTCTTTGACAAAAGATCCCAACTTCACAGCTGCTCTTGCTGCTGCCATTTCTGGGAGATCTGCACAAACCTCAACTGATTAA

> *HmoCYP76AD1*

ATGGATAGCCCAACCCTCTCGCTTTTCATCTTTGCCTCAATCTTTTACTTCATAACTTTTCAAATTGTGAAGCTAGGGTTTAATGTGGTCATGACCTCTAAAAAAACCAAAAGAAGAAGACCCCCCTTACCTCCGGGCCCCAAGCCATTGCCCATCATAGGCAATGTGCTCGAGCTCGGGCCGAAGCCACACCGCTCGTTCGCCGACCTAGCCAAGGTCCATGGTCCACTCATGTCCCTCCGGCTAGGTAGTGTGACCACGATTATCGTGTCATCCTCTGATGTTGCCAAAGAAATGTTCCTTAAAAATGACCAACCCTTGAGCTCCAGCCGAACCATACCCAACTCAGTCACGGCTGGGGATCACCACATGCTGACCATGTCTTGGCTCCCAGTCTCCCCCAAATGGCGGAGTTTTAGGAAGATCACCACCTTCCACCTTCTCTCCCCCCAGCGCCTCGATGCTTGCTCTAGCCTTAGGCAAGCCAAGGTGCAGCAGCTATTCGAGTACGTTCTGGAATGTTCTAGAACCGGCCAGGCCGTCGATATAGGCAAGGCTGCTTTCACGACGTCCCTTAACTTGTTGTCCAAGCTGTTTTTTTCTTTAGAGTTGGCTCACCATAGATCTAGCAAGTCTCAAGAGTTTAAGGACTTAATTTGGGATATTATGGAGGATATTGGGAAGCCTAATTACGCGGATTATTTCCCATGCTTAAAGTACTTTGACCCATGTGGAATACGACGTCGTTTGGCAAATAGTTTTGAGAAATTAATTGAGGTCTTTCAAGGTATTATTCGTCAAAGGCTATCCCTGTCATCTGGCTCTCATACTCATAATGATGTGTTAGATGTTCTTCTTCAATTGTACAACCAAGAGGAACTCACCATGGACGAGATAAACCATCTGCTCGTGGATATATTTGATGCCGGAACAGACACCACTTCCAGTACATTTGAATGGGCCATGGCTGAGTTAATTAAAAATCCGACGATGATGGAGAAAGCTCAAGCTGAAATCAAAGTGGTTCTTGGGAAACAGTCGCATATTCAAGAGTCCGATATCCCAAAATTGCCTTATTTGCGGGCAATTATCAAAGAAACATTGCGTCTACACCCTCCTACTGTATTCCTCCTACCTCGTAAGGCTGAGACCGATGTGGAACTCTATGGCTACACCGTACCAAAAAATGCACAAATACTGGTGAACTTGTGGGCCTTAGGTCGAGACCCCAAAGTTTGGGAAAACCCAGAGGTGTTCTTACCTGAAAGGTTCCTGACTTGCGACATCGATGTTAAAGGAAGAGATTTTGGACTACTGCCTTTTGGGGCAGGAAGGCGAATATGTCCTGGGATGAATTTGGCGTACAGAATGCTGACCTTAATGCTCGCTACGCTTCTACAATCGTTTGATTGGAAACTCCCAAATGAGATGAACTCCAAGAATTTGGACATGGATGAAAAGTTTGGAATAGCATTGCAAAAGACTAAACCCCTTGAAATTATTCCCGTTTGCAAGGATTGA
